# Mechanisms for stabilisation and the maintenance of solubility in proteins from thermophiles

**DOI:** 10.1186/1472-6807-7-18

**Published:** 2007-03-29

**Authors:** Richard B Greaves, Jim Warwicker

**Affiliations:** 1 Faculty of Life Sciences, Michael Smith Building, University of Manchester, Oxford Road, Manchester, M13 9PT, UK

## Abstract

**Background:**

The database of protein structures contains representatives from organisms with a range of growth temperatures. Various properties have been studied in a search for the molecular basis of protein adaptation to higher growth temperature. Charged groups have emerged as key distinguishing factors for proteins from thermophiles and mesophiles.

**Results:**

A dataset of 291 thermophile-derived protein structures is compared with mesophile proteins. Calculations of electrostatic interactions support the importance of charges, but indicate that increases in charge contribution to folded state stabilisation do not generally correlate with the numbers of charged groups. Relative propensities of charged groups vary, such as the substitution of glutamic for aspartic acid sidechains. Calculations suggest an energetic basis, with less dehydration for longer sidechains. Most other properties studied show weak or insignificant separation of proteins from moderate thermophiles or hyperthermophiles and mesophiles, including an estimate of the difference in sidechain rotameric entropy upon protein folding. An exception is increased burial of alanine and proline residues and decreased burial of phenylalanine, methionine, tyrosine and tryptophan in hyperthermophile proteins compared to those from mesophiles.

**Conclusion:**

Since an increase in the number of charged groups for hyperthermophile proteins is separable from charged group contribution to folded state stability, we hypothesise that charged group propensity is important in the context of protein solubility and the prevention of aggregation. Accordingly we find some separation between mesophile and hyperthermophile proteins when looking at the largest surface patch that does not contain a charged sidechain. With regard to our observation that aromatic sidechains are less buried in hyperthermophile proteins, further analysis indicates that the placement of some of these groups may facilitate the reduction of folding fluctuations in proteins of the higher growth temperature organisms.

## Background

The planet Earth offers a rich diversity of habitats, many inhospitable to humans but successfully colonised by other species. Thermophiles are organisms with an optimal growth temperature above 50°C, or above 80°C for hyperthermophiles. In this study we use the terms moderate thermophiles and hyperthermophiles to distinguish organisms within the overall thermophile grouping. Higher temperature habitats require that to function, the organisms living in them express proteins that are intrinsically more thermostable than those from organisms that thrive at lower temperatures. An understanding of the factors that enhance the stability of proteins in extreme conditions is of particular interest because it raises the possibility of engineering enzymes with enhanced high temperature stability and catalytic efficiency for industrial applications. Previous work has addressed this issue, often with confusing and contradictory results.

The simplest of these studies directly compare structures of proteins from thermophilic organisms with those of mesophile-derived homologues [1-12]. However, they tend to lack generality and have led to contradictory suggestions about the factors that are important in enhancing protein thermostability. Other studies use computational methods to compare a greater number of sequences [13,14] or structures, or properties calculated from these [15-19]. Whole genome studies have also been carried out [20-24]. Protein engineering based studies [25-31] have probed thermostability, and attempts to engineer protein stability have been reviewed [32-34]. Some work has combined predictive computation with experimental verification [35,36], whereas other work has concentrated on modelling the anticipated effects of proposed mutations [37].

Many factors have been suggested to play a role in the stability of thermophile-derived proteins. These include ion-pairing [38-40], which was found to be particularly important when occurring in networks [41-43]. The nature and extent of hydrogen bonding is also widely postulated to play a role in the stability of proteins from thermophiles, as is the extent of hydrophobic interaction within the protein.

Other factors that have been examined in relation to thermostability include:

(i) Secondary structure properties, including helix dipole stabilisation [44], the number of residues in α-helical conformation [45], the amount of proline in α-helices [17] and β-strand content [22].

(ii) Protein volume or degree of compactness as well as the number and size of cavities [46,47]. This encompasses measures such as the fractional polar surface area [14], the buried surface area [11], the length of loops [8,12,21,46], and a decrease in the number or volume of cavities within the protein [7,8]. Another study reported that the last feature is not a general correlate of thermostability [9].

iii) Aspects of the general amino acid composition [48]. More specifically a decrease in the number of thermolabile residues e.g. Asn [8], residue hydrophobicity and volume [16], greater number of charged residues [16,18], greater number of β-branched residues [22], greater number of proline residues at compatible sites [49] and fewer polar residues [16,18,22].

iv) The GC content of genes coding for proteins has also been postulated as a possible determinant in protein thermostability although this hypothesis has been refuted [16,21]. Further work indicates that DNA dinucleotide composition correlates with organism growth temperature [50].

Most prominent amongst the listed features are a high degree of optimisation of hydrophobic and charge-charge interactions [51-53]. It has also been suggested that the stability of thermostable proteins may result from a balance between packing and solubility [54]. Enhanced stability of proteins from hyperthermophilic organisms has been discussed in terms of increased rigidity at room temperature [55-58], but this is not universally supported [59,60]. However, there is a consensus view that the enhancement of thermostability in proteins from thermophiles is due to a complex balance of interactions at numerous sites [15,55,61], and that it is difficult to identify a single common determinant [14,62]. It has been proposed that since there is a markedly different temperature dependence of hydrophobic interactions compared to Coulombic interactions, moderate thermophiles and hyperthermophiles should be treated separately for analysis of high temperature adaptation factors [47].

In principle it should be possible to use knowledge of factors that predispose a protein toward enhanced thermostability to predict which mutations may be good targets for protein engineering. Thus there have been attempts to predict mutations to enhance protein thermostability [63,64]. There also exist web resources such as FoldX [65] and PoPMuSiC [66] for the prediction of stability changes upon mutation.

The current study tests several properties for their ability to discriminate between datasets of 291 protein structures from thermophiles, and their closest counterparts amongst mesophile protein structures. We use electrostatic calculations to quantify predictions of charged group contribution to stability, and estimates of sidechain rotamer entropy to examine questions related to packing, as well as presenting computations of a number of other features. We find, as expected, that charge interactions are key discriminators, but unexpectedly our calculations suggest that while the number of charged groups and their contribution to folded state stability are both important, these two aspects do not correlate. The change in the number of charged groups is consistent with a role in preventing protein aggregation. A further novel result is the finding that aromatic sidechains are somewhat less buried in proteins from hyperthermophiles. This may indicate a role in mitigating against fold fluctuations at higher temperatures.

## Results

### Thermophile protein structures

Our datasets containing 291 protein chains from thermophiles and 272 unique protein chains from mesophiles are considerably larger than those used previously in computational analyses of structure and thermo-adaptation. Of the 291 chains from thermophiles, 144 derive from hyperthermophiles and 147 from moderate thermophiles. Smaller datasets of 67 thermophile proteins (30 from hyperthermophiles and 37 from moderate thermophiles), and the matching mesophile proteins, are formed with the conditions of pair E-value < 10^-2 ^and chain length difference ≤ 30 amino acids. These latter sets represent an attempt to focus on pairs consisting of homologous proteins, and to remove any systematic bias arising from chain length variation in organisms. For convenience we refer to the '291' and the '67' sets.

### Calculated ionisable group contribution to folding free energy

**Figure 1:**
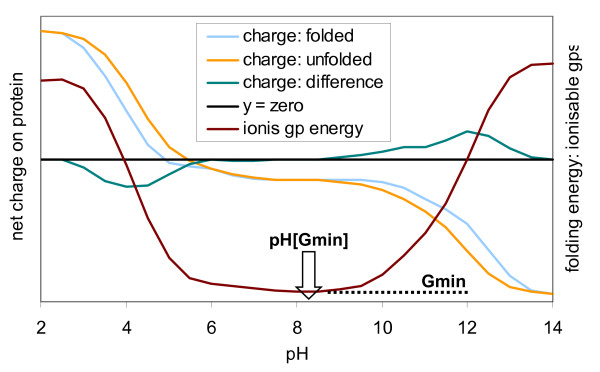
Schematic diagram of pH-dependent properties that can be predicted: titratable charge for folded and unfolded forms; the difference of these determines the pH-dependence of folding free energy (due to ionisable groups). Gmin is the minimum value of this energy, at pH [Gmin].

**Figure 2:**
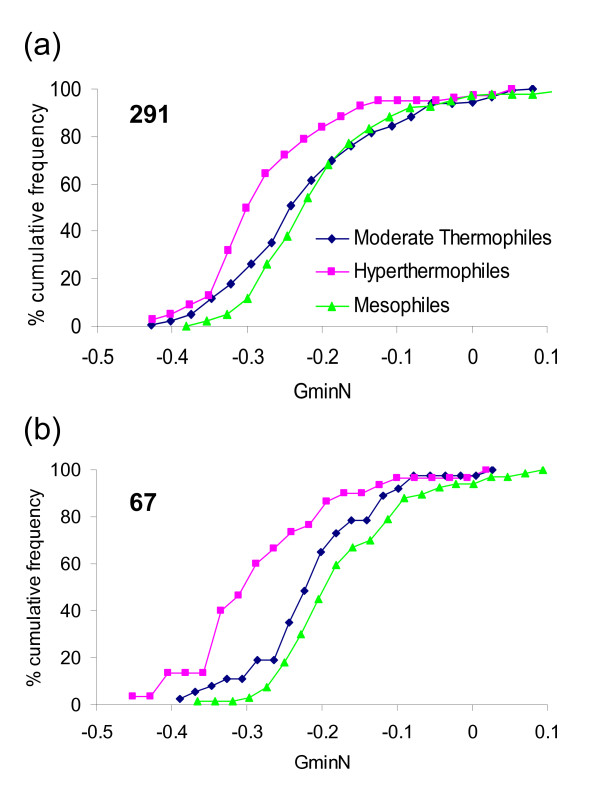
Separation by GminN. Cumulative frequency distributions of GminN calculated for each protein in the dataset and grouped according to origin as mesophile, moderate thermophile or hyperthermophile. (a) The 291 set. (b) The 67 subset of homologous pairs with E-value < 10^-2 ^and a chain length difference of less than 30 residues.

The minimum of the curve describing the pH-dependence of ionisable group contribution to the free energy of folding (Gmin, Figure 1), divided by the number of residues in the chain (GminN), was examined. Normalisation was performed to reduce the effect of length differences between proteins. Cumulative frequency distributions of GminN are separated for both the 291 sets and 67 subsets of hyperthermophile- and mesophile-derived proteins (Figure 2a,b), reflecting the anticipated greater contribution of charged group interactions to the free energy of stabilisation for proteins from hyperthermophiles. We also explored the ionisable group contributions at notional pH extremes that relate to full protonation or full deprotonation, again normalised by the protein length. Although some separation in the curves for the hyperthermophile and mesophile sets was observed (not shown), these appeared to recapitulate the GminN result and were not analysed further.

### Entropy associated with sidechain rotamers and amino acid composition

**Figure 3:**
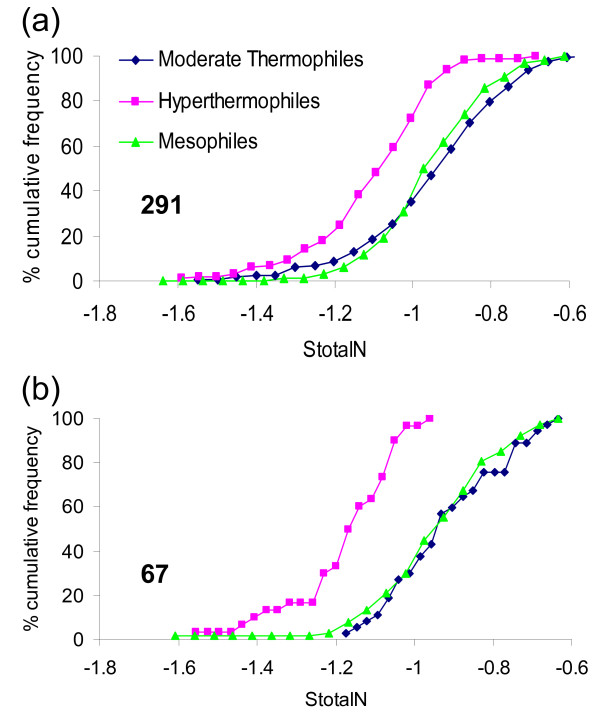
Separation by StotalN. Cumulative frequency distributions of StotalN. (a) The 291 set. (b) The 67 subset.

**Figure 4:**
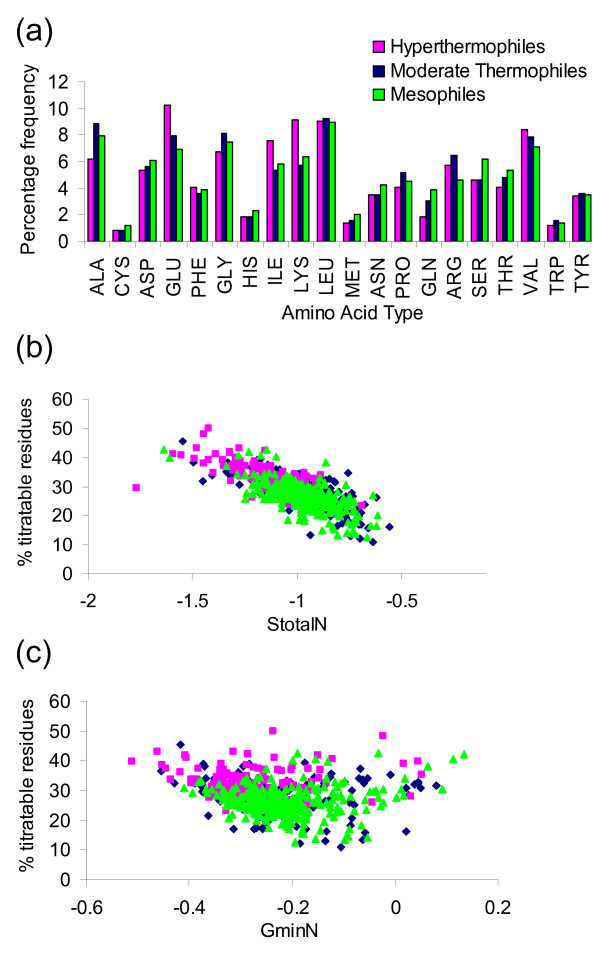
Amino acid composition, GminN and StotalN. (a) Composition of proteins in the 291 set. Correlation of the percentage of titratable residues per protein with (b) StotalN, and (c) GminN.

We studied the entropy for all sidechain rotamers, given complete conformational freedom, and normalised by the number of amino acids, StotalN. This property relates to amino acid composition since it is not affected by protein conformation. The cumulative distributions of StotalN values show separation for hyperthermophile proteins compared to mesophile proteins (Figure 3a,b). It is known that amino acid composition varies between proteins from thermophiles and mesophiles. Figure 4a shows this for our dataset, in particular a higher proportion of charged and longer sidechains in thermophile proteins relative to mesophile proteins, which is particularly evident in the subset of 144 hyperthermophile proteins.

We sought to establish whether an overall increase in the number of ionisable residues underpinned our observations for StotalN. There is a correlation between StotalN and the overall percentage of ionisable residues that are likely to carry net charge at neutral pH (Figure 4b). However there is not clear correlation between GminN and the percentage of ionisable residues (Figure 4c) or between GminN and StotalN (not shown). This observation implies that enhanced stabilisation of the folded state for thermophile proteins results from the 3D arrangement, rather than the number, of charged groups [40].

### Sidechain rotamer restriction in the folded state

**Figure 5:**
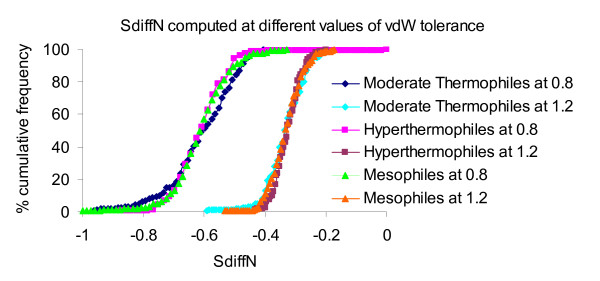
Lack of separation by SdiffN. SdiffN is shown for mesophile, moderate thermophile, and hyperthermophile proteins, at two values of the van der Waals tolerance parameter (used in sidechain packing), 0.8 and 1.2 Å.

The quantity SdiffN is the (protein length normalised) difference between StotalN and the fold-restricted case, estimated from mean field calculations of rotameric restriction in the folded state. As such, SdiffN is a measure of sidechain 'lock down' in the folded state of the protein. SdiffN was not a useful discriminator between proteins from moderate thermophiles or hyperthermophiles and mesophiles (Figure 5). These calculations are affected by the van der Waals tolerance allowed for atom clashes in sidechain packing. A value of about 0.8 Å is generally required to pack back the experimentally-derived rotamers, relating to overlap required for some interactions in a United Atom model. Calculation of SdiffN was repeated for several values of clash tolerance (0.4, 0.8, 1.0, 1.2, 1.4, 1.6 and 2.0 Å). The best discrimination of SdiffN distributions was apparent for the tolerance parameter set to 1.2Å (Figure 5). We interpret SdiffN as related to conformational flexibility, for sidechains, so that the current result is roughly in accord with the observation [48] that any increase in sidechain flexibility in thermophile proteins compared to mesophile proteins is small. It has been hypothesised that the basis for thermophile proteins containing a greater proportion of Lys over Arg, is a difference in the number of accessible rotameric states [48]. In a subsequent section we look at variations in dehydration energy that could contribute to changes in the percentages of charged residue classes.

### Contact order and amino acid packing

**Figure 6:**
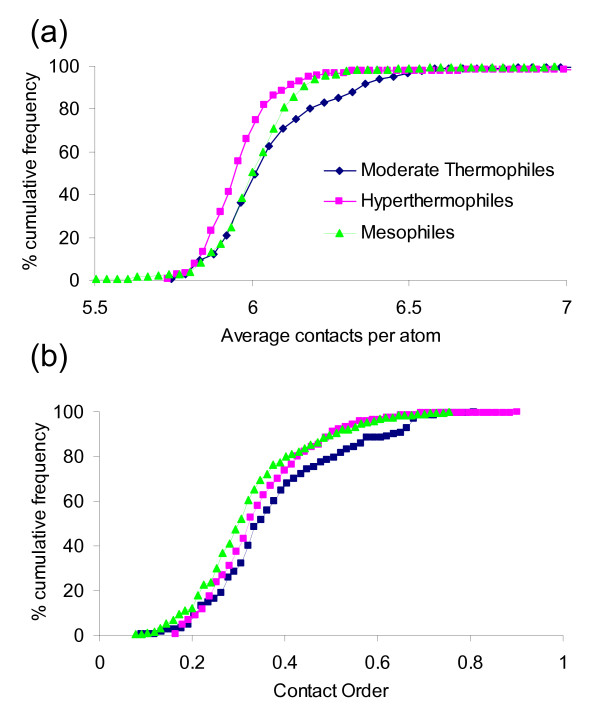
Separation by degree of compactness. (a) Packing calculated with the average number of contacts per atom. (b) Packing calculated using contact order [67].

The cumulative distribution for the 291 set shows lower numbers of contacts per atom for hyperthermophile proteins relative to mesophile proteins, and slightly larger for proteins from moderate thermophiles (Figure 6a). Using relative contact order [67], the relative ordering of cumulative distributions changes for the 291 set, which shows only small differences between the datasets (Figure 6b). These results contrast with previous work [68] that found contact order strongly discriminated enzymes from the hyperthermophile *T. maritima *and homologues from mesophiles.

### Charged group desolvation energy

**Figure 7:**
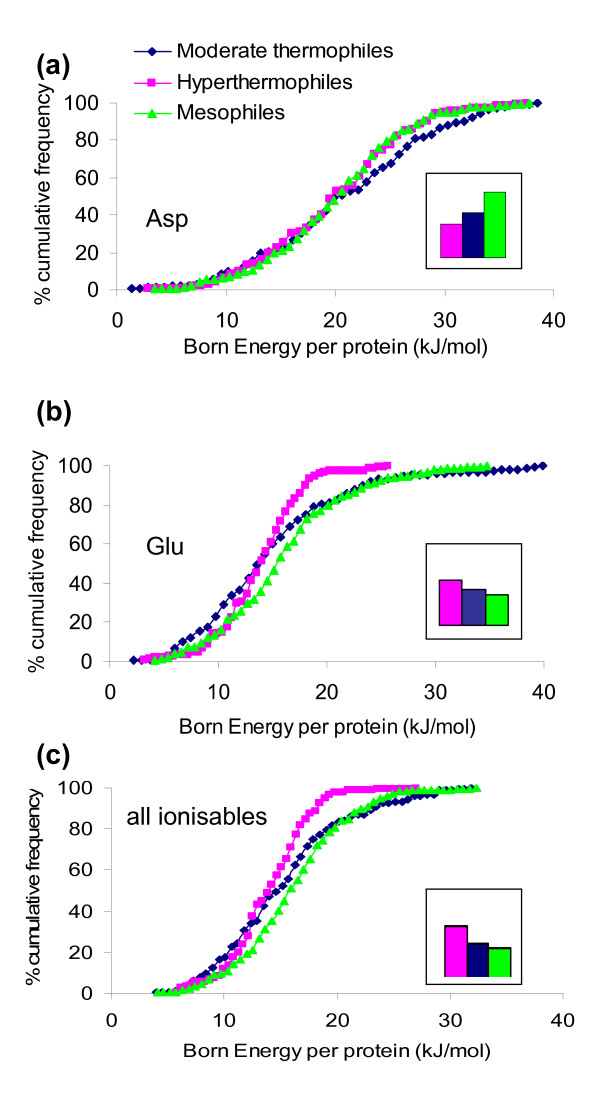
Separation by Born (desolvation) energy of ionisable groups, summed over each protein. (a) Aspartic acid. The inset in this and other panels shows the relative composition for the given amino acid(s). Thus aspartic acid is most common in mesophiles and least in hyperthermophiles. (b) Glutamic acid. (c) All ionisable groups likely to be charged at neutral pH.

The GminN analysis looked at charge-charge interactions with a simple Debye-Hűckel (DH) model that neglects desolvation energies. A Finite Difference Poisson-Boltzmann (FDPB) calculation was used to estimate dehydration energies for ionisable groups likely to be charged at neutral pH. It was found that hyperthermophile and mesophile proteins are differentiated by the Born energy summed over all titratable groups that are likely to carry net charge at neutral pH (Figure 7c). This differentiation was principally due to Glu, Lys and Arg, and generally relates to more solvent exposure in the folded form. For example, Asp possesses a shorter sidechain (Figure 7a) and is less able to achieve the same level of solvent exposure as Glu (Figure 7b). It can be seen that overall Born energy is lower for Glu than Asp and lower still in hyperthermophiles. Aspartic acid sidechains presumably are unable to adapt conformationally to reduce Born energy, consistent with their substitution by Glu residues in thermophiles (particularly hyperthermophiles, note the relative abundance histograms in Figure 7). Individually these energy components are relatively small, but are more significant summed over a protein. We are able to rationalise changes between amino acid compositions in energetic terms (e.g. Glu for Asp), but this desolvation argument does not account for the overall increase in ionisable groups. This is investigated (in later sections) in terms of protein solubility, i.e. differences between folded and aggregated states rather than between folded and unfolded states.

Various Asp/Glu substitutions have been studied in *E. coli *and *M. jannaschii *thioredoxins [69]. It was found that generally Asp for Glu substitutions stabilised a protein, and without obvious environmental or salt-bridging differences, this was attributed to a higher conformational entropy for Glu relative to Asp. The current work indicates that a further possibility should be considered, the increased length of the Glu sidechain allowing for relatively more hydration, giving a lower desolvation penalty upon protein folding.

### Surface area properties

No clear separation was observed between the cumulative frequency plots of the ratio of polar to non-polar surface area in thermophile and mesophile proteins (not shown), where polar area includes charged atoms from groups that are net neutral and net charged. The ratio was about 0.8 at the 50% point of all cumulative distributions. Thus any increase in the hydrophobic effect at raised temperature does not lead to an alteration in overall non-polar surface area. We wondered whether there may be, within the overall measure, a difference in non-polar patch size at the upper extreme. Taking 6 Å radii around each group centre, the non-polar surface area within each patch thus defined was calculated. This also gave negligible separation (not shown), rather than the large change that might have been expected if the temperature-dependence of non-polar interactions was closely coupled to aggregation. The result was uniform over several choices of patch radius, in accord with previous work [47].

**Figure 8:**
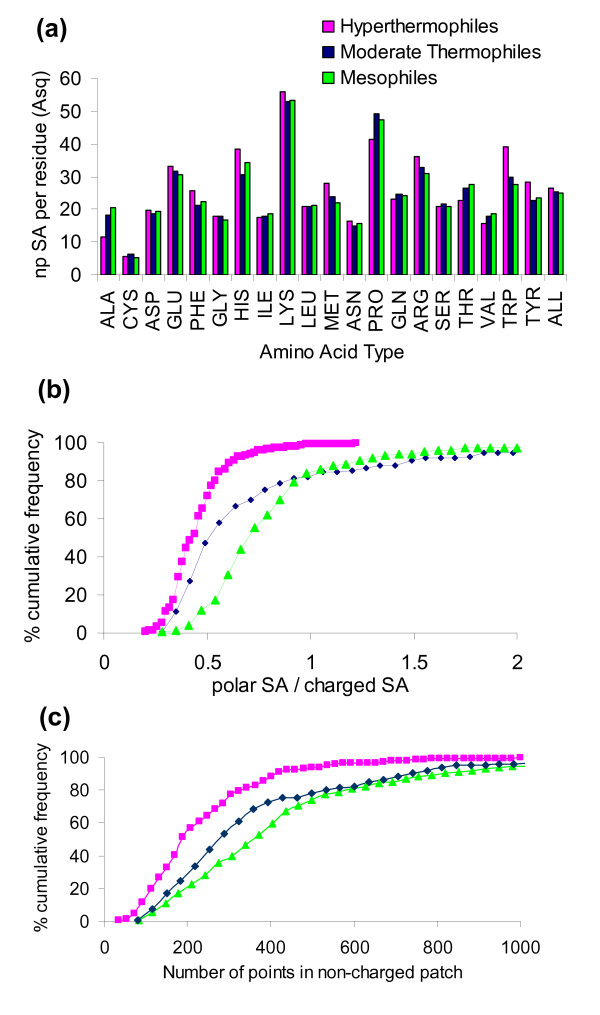
Accessible surface area properties. (a) Non-polar solvent accessible surface area per residue for each residue type. (b) Separation by cumulative distributions of the ratio of polar to charged solvent accessible surface area for each protein in the 291 set. (c) Separation by the size (number of points) of the largest patch that does not contain a group bearing net charge.

Next we looked at the distribution of non-polar surface area by residue type (Figure 8a). As expected from the overall results of roughly uniform non-polar area, there are counteracting behaviours. Amino acids with notable falls in non-polar surface area, mesophiles to hyperthermophiles, are Ala and Pro, whilst residues going in the opposite direction include Phe, Met, Trp and Tyr. The relative burial of Ala and Pro in hyperthermophiles is allied to zero sidechain entropic cost, and thus may represent a folded state stabilisation mechanism. Such behaviour is generally associated with aromatic residues (Phe, Trp, Tyr), and yet we see that they expose more non-polar surface, on average, in hyperthermophile proteins than in mesophile proteins.

The overall increase of charged groups in thermophile proteins is evidenced by the well-known increase in surface area associating with net charge in comparison to that due to dipolar groups (Figure 8b), also known as *CvP*-bias (charged *versus *polar/non-charged) [24]. In order to probe the distribution of charged residues, we used a surface grid system that was previously developed for functional site identification [70]. Each ionisable group centre became the origin of a hydration sphere. With hydration spheres superposed on the surface grid, we recorded grid patches covering areas that were outside hydration shells. The largest 'non-charged' patch for each protein was used in cumulative frequency distributions (Figure 8c). It is clear that not only do hyperthermophile proteins generally have more groups bearing net charge, but also they are located such that the largest surface patches without these groups are smaller than in mesophile proteins. Therefore, the temperature-related differences in numbers of groups bearing net charge, that do not directly correlate with the GminN contribution to thermostability, relate to a manipulation of protein surfaces that is consistent with the prevention of aggregation.

### Distinguishing thermophile proteins from mesophile proteins

**Figure 9:**
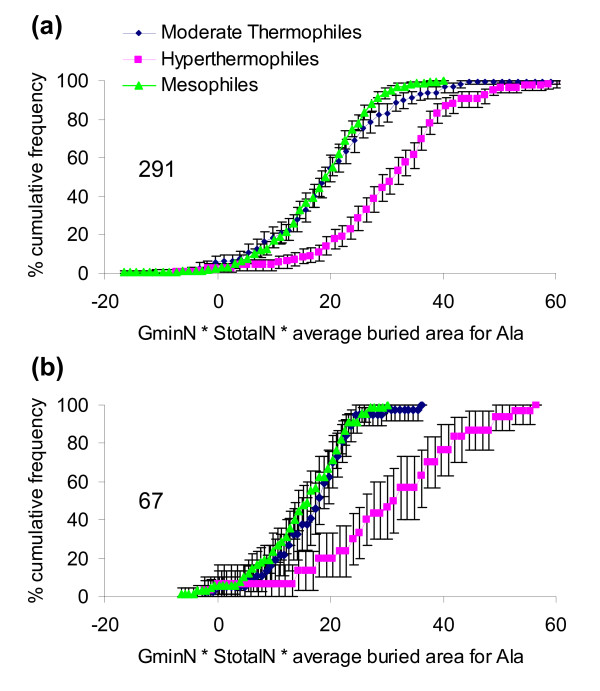
Separation of proteins by three properties. The triple product GminN * StotalN * (the average buried area per alanine residue) is used for (a) 291 set and (b) the 67 set. Error bars are derived from the 5 and 95 percentile levels by bootstrap resampling.

Our observation of the lack of correlation between GminN and StotalN implies that thermophile-mesophile protein discrimination will improve with their combination. We plotted the triple product GminN * StotalN * (100 - % of Ala non-polar surface area), so that the third component increases with Ala burial (Figure 9a,b). This follows the observation of substantial changes in the surface area properties of several residues, in the different datasets. Alanine was chosen since it is a relatively common residue, with data available for all proteins. The triple product is a good discriminator, particularly for the smaller, length restricted, datasets.

### Thermophile-mesophile protein homologue pairs

**Figure 10:**
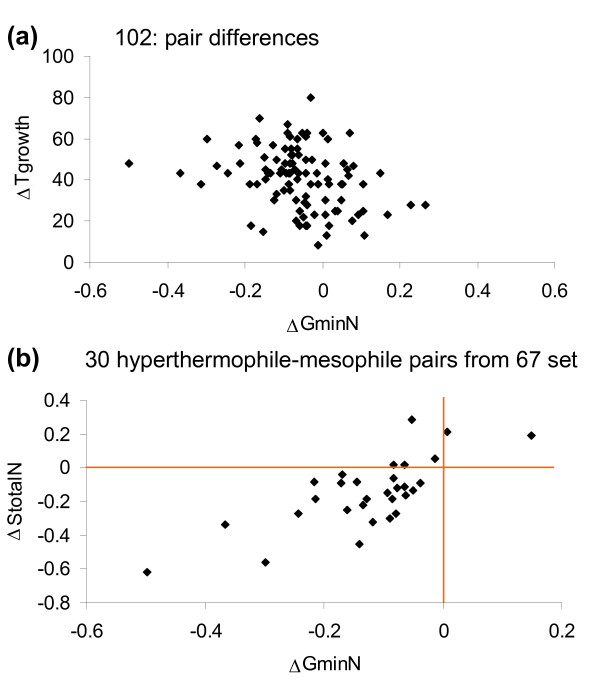
Differences within homologous pairs. (a) Correlation between the difference in growth temperature and the difference in GminN for the 102 thermophile-mesophile pairs in the 291 dataset that have an E-value < 10^-2^. (b) Correlation between the difference in GminN and the difference in StotalN for the 30 hyperthermophile-mesophile pairs in the 67 dataset. Lines of zero ΔStotalN and ΔGminN are marked.

Analysis of ΔGminN and ΔTgrowth for 102 homologue pairs (pairs from the 291 sets with E-value < 10^-2^) showed no detailed correlation between these quantities (Figure 10a), despite the moderate separation between hyperthermophile and mesophile protein datasets given by GminN in Figure 2. The result from Figure 2 is evident in the relatively low population of points at higher ΔTgrowth and positive ΔGminN i.e. hyperthermophile proteins generally have lower GminN than mesophile proteins. We presume that since the members of each thermophile-mesophile protein pair in Figure 10a are evolutionarily separated, the many changes in various contributions to protein stabilisation will swamp the overall drift in GminN values.

When differences between the 30 hyperthermophile-mesophile protein pairs of the 67 set (E-value < 10^-2 ^and restricted chain length difference) are examined (Figure 10b), some correlation of ΔGminN and ΔStotalN is apparent. This is partly due to the extreme values where a particularly large change in GminN accompanies a large change in StotalN. At lower values of the differences, a large spread remains. It is notable that the vast majority of these 30 pairs exhibit decreased/stabilising GminN and more sidechain rotamers (decreased StotalN) on moving from mesophile to hyperthermophile proteins.

### Charge-charge interactions and protein stability

**Figure 11:**
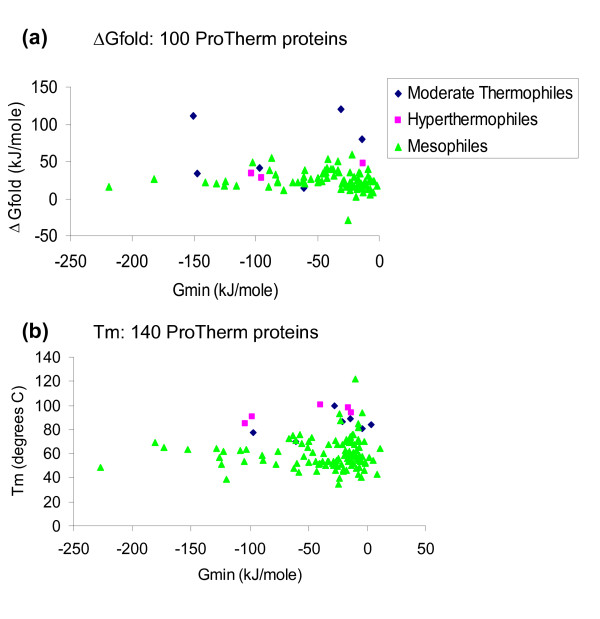
ProTherm data, and calculations. (a) Scatter plot with ΔG_fold _for 100 proteins in the ProTherm database and calculated Gmin. Whereas ProTherm records ΔG_fold _as more positive for a more favoured folded state, Gmin calculations are in the opposite sense. (b) Scatter plot with the melting temperatures for 140 proteins in the ProTherm database and calculated Gmin.

Given that we have a collection of properties that provide some distinction between proteins from organisms at different growth temperatures, we looked also at proteins for which stability data (ΔG_fold _and/or T_m_) are available in the ProTherm database [71]. Experimental ΔG_fold _or T_m _are plotted against the calculated Gmin (Figure 11a,b). A large majority of the ProTherm proteins are from mesophiles. There is no correlation between our calculated charge-charge interactions and stability, using either the computed values per protein (Gmin) or the values per amino acid (GminN, not shown). This result emphasises that protein stability is a complex mixture of components, any one of which will not necessarily be a reliable indicator. Charge-charge interactions contribute to separation of thermophile and mesophile proteins in our analysis, but not to separation within a mesophile set, indicating that organism growth temperature is an important factor.

## Discussion

The current study uses a large sample of proteins from thermophiles and mesophiles to compare physical characteristics. Some of the quantities investigated have proved to be useful discriminators of proteins, whereas others have not. This information is summarised in Table 1, with reference to the relevant Figure panels, listing of the values for cumulative distributions at the 50% level, and the results of t-test comparisons between proteins in the mesophile, moderate thermophile and hyperthermophile sets. We now discuss the properties in the following broad categories: amino acid composition; packing; charge interactions; surface properties; with a final section discussing the relevance of the current study to protein thermostability.

### Amino acid composition

The greatest difference in amino acid composition between mesophile and hyperthermophile proteins was their proportion of titratable residues (Figure 4a), being higher for hyperthermophiles [16,18,22], with the largest changes for Glu and Lys [48,69]. We see a small decrease in the proportion of Asn [8], in common with other polar residues that do not carry net charge. Consistent changes in the proportions of β-branched residues [22] between mesophile and thermophile datasets were not clearly apparent (apart from a slight increase in the proportion of isoleucine observed in hyperthermophile proteins), nor was there evidence for a substantial shift in the proportion of proline, that had been reported previously [49]. Relative proportions of hydrophobic residues in thermophile and mesophile proteins [16] do not show a clear trend in our study (Figure 4a).

In overall terms, amino acid composition for proteins from higher growth temperatures shows a trend for more ionisable groups, compensated by less polar, non-ionisable groups, with relatively little change in non-polar amino acids.

With regard to GC content of genomes, although there has been some report of a correlation to organism growth temperature [72], most studies of this property fail to find any such correlation [16,21,24,73-76]. We therefore did not investigate this factor any further. Neither did we examine dinucleotide composition, which is a promising correlate of organism growth temperature [50].

### Packing in folded proteins

Contrary to earlier results [68], only a weak correlation between relative contact order and thermophile/mesophile origin was found in our sample of proteins. Although different datasets could contribute to the discrepancy, it is also possible that protein compactness is not a major determinant of thermophile compared with mesophile proteins [15]. It has been reported that proteins from hyperthermophiles are more stable than those from mesophiles in part because they are more rigid at room temperature than the mesophile proteins. The current study employed the quantities StotalN and SdiffN to represent the flexibility of sidechains summed over free amino acids, and the differential in sidechain flexibility upon folding, respectively. StotalN was found to be a good discriminator between hyperthermophile and mesophile proteins (and correlated with ionisable group composition, via the number of rotatable bonds). However, SdiffN was not a good discriminator. It has been suggested that the increased entropy for a greater number of accessible rotameric states for lysine as compared to arginine in a similar environment, might explain the greater increase of lysine numbers over arginine in hyperthermophile proteins as compared to mesophile proteins [48]. The current study identifies the increase of lysine numbers in hyperthermophile proteins, but since the overall SdiffN parameter is a poor discriminator, it does not support the argument that sidechain restriction is a key factor.

Although our measures of packing and rigidity do not substantially separate hyperthermophile and mesophile proteins, such properties may still be relevant for sub-groupings, and particularly it does not necessarily follow that thermostability cannot be engineered along these lines. For example, increased thermostability has been achieved with improved packing of the hydrophobic core [29], whilst stabilisation has also been engineered via the introduction of proline residues to decrease sidechain entropy in the folded state [26].

### Charge interactions

We see three overall trends associated with charged residues: (i) As anticipated, the electrostatic component of the free energy of folding, GminN, separates thermophile from mesophile proteins, (ii) Our measure StotalN contributes to separation of thermophile and mesophile proteins, and correlates with percentage of ionisable groups, (iii) Within the overall change in ionisable group composition, there are compositional swaps between Glu and Asp, and Lys and Arg.

Taking issue (iii), the average desolvation energy for all titratable groups was higher in mesophile proteins than in thermophile proteins. Therefore, these residue types are not only more common, but also less buried on average in thermophile protein structures than mesophile. In energetic terms our calculations suggest that thermophile proteins reduce the energy penalty associated with any partial burial of groups bearing net charge. This reasoning would explain the compositional swaps, e.g. Glu has a longer sidechain than Asp and can attain higher solvent exposure more readily, and is a potential explanation for Lys/Arg alterations [48]. It is also consistent with a study of the temperature-dependence of desolvation and charge-charge interaction components of salt-bridges [77].

**Figure 12:**
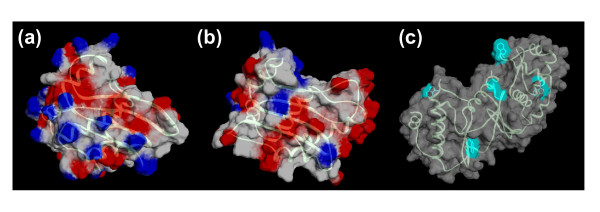
Surface adaptation in hyperthermophiles. Numerous surface acidic residues (red) in both hyperthermophile, (a) 1oz9, and mesophile, (b) 1xax, members of a homologue pair, are joined by many more basic residues (blue) in the hyperthermophile representative. (c) The location of tryptophan residues (cyan) in a hyperthermophile protein (pdb id 1zar).

With regard to GminN, one might have expected, given the large number of proteins in the current study, that some correlation between GminN and the proportion of ionisable groups would be evident. However, this was not the case in comparisons of GminN and StotalN (which itself correlates with the proportion of ionisable groups). It is therefore, generally, the relative spatial arrangement of the charged groups rather than their numbers that is a determinant of thermostability. When hyperthermophile-mesophile homologue protein pairs are studied in the 67 set (with the restraint of similar chain lengths), some relationship between the pair differences ΔGminN and ΔStotalN is observed, most clearly for pairs with large differences. An example of such is shown in Figure 12a,b. The hyperthermophile protein has 32/26 basic/acidic residues, compared with 13/31 for the mesophile protein. This addition of positive charge in the hyperthermophile protein drives ΔGminN and ΔStotalN. For a potential molecular explanation of the general discriminating power of StotalN, we turn towards surface features and propose a link with avoidance of aggregation.

### Surface properties

Whereas a previous report [14] found fractional polar surface area to be a possible determinant of thermostability, we find that a comparable measure (the ratio of polar to non-polar accessible surface area) did not discriminate thermophile and mesophile proteins. Further, there is only a small difference in the distributions for the largest non-polar surface patch, being slightly larger on average in mesophile proteins. Possibly this relates to an offsetting of non-polar patches becoming stickier and more susceptible to mediating non-specific aggregation at higher temperature, but the overall effect is small.

Examination of the polar and non-polar surface areas for each of the twenty amino acid types revealed that Ala and Pro showed a large drop in average surface area i.e. tended to become more buried in hyperthermophile proteins, whereas Phe, Trp, Tyr and Met all showed a rise i.e. became less buried. In terms of temperature-driven entropic effects, these observations make sense in relative terms. Pro and Ala, each with fixed configurations, are burying more non-polar area for no additional sidechain restriction in thermophile proteins. However, it is generally thought that the larger non-polar sidechains are ideal candidates for forming the folding core of a protein. It is therefore a surprise that they are more exposed in hyperthermophile proteins. Figure 12c shows the hyperthermophile-derived member of the protein pair in the 67 set that has the largest change in tryptophan burial. A number of Trp residues are located towards the surface but are still mostly buried, (there is also one more exposed Trp sidechain). Several of the mostly buried Trp residues are located towards the end of secondary structural elements. We speculate that such residues may be located to resist partial unfolding or fraying of secondary structure elements, which may require more regulation at higher temperatures.

The size of the largest non-charged surface patches (regions lying between residues bearing net charge), inversely correlated with the proportion of titratable residues and StotalN, so that the overall increase in numbers of ionisable groups in thermophile proteins (particularly in hyperthermophile proteins) carries over to their coverage across the entire surface. Recalling that the distribution of non-polar patch size does not vary substantially between proteins from mesophiles and from thermophiles, one interpretation of our results is that the location of groups bearing net charge, rather than dipolar groups, mitigates against non-specific aggregation. We hypothesise that the enhanced hydrophobic effect at higher temperatures, that will drive associations and could lead to aggregation, are counteracted by a larger population of groups bearing net charge that resist dehydration and aggregation processes. However, hyperthermophile proteins do not separate from mesophiles entirely when using StotalN, percentage of ionisable groups, or non-charged patch size, indicative of other mechanisms contributing to changes in protein solubility. This more complex picture is consistent with the finding that a set of 30 proteins was split roughly in half according to whether solubility increased or decreased with temperature over the range 4–45°C [78].

### Protein folded state stability

Although GminN contributes to the separation of mesophile and thermophile proteins, our examination of stability data in the ProTherm database showed that it did not correlate to ΔG_fold _or T_m_. The ProTherm data are mostly of mesophile origin, so there is a difference between testing correlation with Gmin for proteins that have evolved to function at different temperatures, and those that function in a narrow temperature range, but exhibit variation in folded state stability. Presumably Gmin and GminN are poor indicators of ΔG_fold _since, although many studies show that stability can be modulated by alteration of charge interactions, overall contributions vary considerably between mesophile proteins, and several factors together determine protein stability. Our study supports the idea that the enhanced stability of thermophile proteins is also a balance of factors [43,55]. However, in adjusting between mesophile and thermophile growth temperatures, particular use is now made of charge interactions [40,79]. According to our calculations this applies to desolvation energy as well as charge-charge interactions. It is possible that the temperature-dependence of the water dielectric response plays a significant role in these observations. DH charge-charge interaction and FDPB desolvation energy calculations, for all proteins, were made with a relative water dielectric of 78.4, corresponding to 25°C. This value falls, for example, to 66.8 at 60°C [80], giving a substantial increase for water-dominated charge-charge interactions. The relative change in desolvation energies will be less over this temperature range, since in rough terms these vary according to (1/ε_protein _- 1/ε_water_), where ε_protein _is about 2–4 (4 in our FDPB calculations). Nevertheless the change will be to make desolvation less unfavourable at higher temperature, supporting the suggestion that interactions involving groups bearing net charge are well-suited for relative stabilisation of folded protein structure at higher organism growth temperatures [81].

The average degree of stability enhancement that we predict for charge interactions can be approximated from the cumulative distributions. For hyperthermophile proteins relative to mesophile proteins (Figure 2a) we see at the 50% cumulative ordinate a difference of about 0.1 kJ/mole per residue. For a 200 residue protein this is about 20 kJ/mole, a significant fraction of the range of differences shown in measurements of protein stabilities [82]. This estimate neglects the enhancement of such interactions due to the temperature-dependence of water dielectric. One factor not included is the effect of residual charge-charge interactions in the unfolded state [83-86]. These tend to reduce predicted ΔG_fold_. However, our emphasis is on the calculated GminN as a discriminator of thermophile and mesophile proteins, rather than as a direct measure.

The properties of proteins from moderate thermophiles are generally closer to those of proteins from mesophiles than to proteins from hyperthermophiles. This behaviour may represent complexity of the underlying molecular details of temperature-dependence, as well as combination of different features. We have hypothesised that desolvation energy changes are mediated by (small) alterations of water exposure as well as swapping amino acid type within basic or acidic groups. Over a range of growth organism temperatures, one property may be saturated before another. For example, the calculated desolvation energy for Arg is about equal for hyperthermophile proteins and moderate thermophile proteins, both separated from mesophile proteins, whilst Arg composition peaks at moderate thermophile proteins and then decreases as the growth temperature increases further. Thus we see some evidence that hyperthermophile proteins and moderate thermophile proteins may be stabilised via a different balance of mechanisms [47].

## Conclusion

We have calculated various properties for datasets of thermophile and mesophile proteins. Since we were unable to find structures for mesophile protein homologues of all 291 thermophile proteins, results have been compared between the full 291 sets of proteins and 67 protein pairs with lower E-value and similar chain lengths. The overall results are similar in that a separation in the 67 analysis corresponds to a separation in the 291 data (compare Figure 2a,2b; Figure 3a,3b; Figure 9a,9b). Our studies support the conclusion that no property correlates universally with hyperthermostability [47,55]. Even for predicted ionisable group contribution to stability, which is one of the few properties tested that gave substantial discrimination, it does not transfer to a correlation with thermostability data for mesophiles in the ProTherm database. Our results concur with the view that folded state stability is a complex mixture of factors. The fact that GminN is a significant factor in the current study indicates that the temperature-dependence of water dielectric plays a role in elevating the importance of charge interactions for proteins from thermophilic organisms.

A less expected result in our work was the lack of correlation between the well-known increased proportion of ionisable groups in hyperthermophile proteins, and GminN. This increase carries over to a size decrease in the largest non-charged surface patches, (patches not containing a net charge), and may be the signature of a mechanism to prevent aggregation, based on dehydration penalty, that is enhanced at higher temperatures. Non-polar patches themselves do not appear to change geometry greatly between proteins from thermophiles and mesophiles. Studies of aggregation related to misfolding [87] invoke charged residues as 'gatekeepers', flanking β-strands that would otherwise be prime candidates for seeding amyloidosis in misfolded proteins [88], an observation related to a recorded propensity for capping exposed β-strands in folded proteins with charged residues [89]. Our work suggests that specific placement of charges to prevent aggregation of folded proteins may be an important factor, evidenced by the separation of mesophile and hyperthermophile proteins.

A common theme that we observe is that whereas a variety of mechanisms influence protein stability and solubility, a subset may be best placed to modulate differences over the mesophile to hyperthermophile temperature range. Thus charge interactions appear to be important for stability and solubility. Perhaps our most surprising observation is that large non-polar sidechains are somewhat more exposed in hyperthermophile proteins, leading us to speculate on a role in suppressing unfolding fluctuations at higher temperature.

## Methods

### Datasets of extremophile and mesophile protein structures

Starting from the November 2005 release of the RCSB [90], structures solved at a resolution worse than 2.5Å, as well as oligonucleotide, carbohydrate and totally synthetic structures, were removed. Using the PDB *source.idx*, each PDB entry was assigned a species of origin and these were then classified according to their ambient habitat as thermophilic, psychrophilic, acidophilic, alkalophilic, halophilic or thermotolerant, psychrotolerant, acidotolerant, alkalotolerant or halotolerant. Organisms that were mesophilic and neutrophilic were removed from the dataset completely. Higher organisms were not classified for tolerances, since they can be heat or cold tolerant by mechanisms that shield cells from the environmental temperature.

The classification of organisms was based on searching for each organism name in conjunction with any of the following terms: thermophile; thermophilic; "heat tolerant"; thermotolerant; acidophile; acidophilic; "acid tolerant"; acidotolerant; halophile; halophilic; "salt tolerant"; halotolerant; psychrophile; psychrophilic; "cold tolerant"; psychrotolerant; alkalophile; alkalophilic; "alkali tolerant"; alkalotolerant; alkaliphile; alkaliphilic; alkalitolerant. Results were cross-referenced with specialised web sites such as, the *List of Prokaryotic Names with Standing in Nomenclature *[91] to provide additional insight into the preferred habitats of the organisms of interest. In a few cases, organisms with growth temperatures down to 45°C were identified as thermophilic, and proteins from these organisms were retained in our analysis.

These classifications were used to extract subsets, and the data culled at 25% sequence identity using the PISCES server [92] with default parameters. Subsets were further reduced by eliminating oligomeric entries using the Protein Quaternary Structure server and the associated list of biological units [93]. BLAST [94] searches against the PDB were used to find possible homologues of the remaining thermophile protein entries, which were then checked to see if they were from non-extremophiles and were monomeric proteins. The top ranking (by E-value) protein was chosen from each BLAST search unless another protein with similar E-value more closely shared the function of the search target. In this manner we derived a set of 291 thermophile and mesophile protein pairings, where a few structures were removed as unsuitable for calculation, for example those with only C_α _coordinates. Due to some residual redundancy, we actually find only 272 mesophile proteins, since some match to more than one thermophile protein. It is important to note that some of these pairings are not homologous proteins, since definite mesophile protein homologues could not be found for all the thermophile proteins (confirmed by closer inspection of higher E-value representatives). We label these the '291' sets, with roughly equal numbers of thermophile and mesophile proteins. These are used for comparisons that do not depend on strict pairings, and which form the bulk of our analyses. Within these sets, 102 pairs are related by BLAST E-values of < 10^-2^, and 70 pairs < 10^-10^. Where seeking to supplement analysis of the 291 sets, with probable homologues of similar size, we used the '67' sets, formed from those 67 of the 102 pairs (E-value < 10^-2^) that have chain lengths differing by ≤ 30 amino acids. Of these 67 pairs, 37 contained proteins from moderate thermophiles, and 30 contained proteins from hyperthermophiles. The complete datasets are described in Additional file 1.

### Calculation of electrostatic properties

The 291 sets of thermophile and mesophile proteins were processed for various computed electrostatic and sidechain entropy properties, with a handful of pairs omitted due to failures from problems such as encountering C_α_-only structures. Electrostatics calculations used the Debye-Hückel method to study the pH-dependent contribution due to ionisable groups, a model suitable for the vast majority of such groups located at the protein surface with water-dominated charge-charge interactions [86]. In this work we refer to this pH-dependent contribution due to ionisable groups as charged group interactions, for brevity. The relative dielectric was 78.4 and ionic strength 0.15 Molar. Monte Carlo sampling generated the ionisation status over the pH range [95], from which the pH-dependent energy could be calculated. This property was converted to an absolute value by addition of the ionisable group charge-charge interaction free energy computed at an extreme (low) pH value, corresponding to full protonation [96]. Figure 1 shows a schematic plot for these results, labelling the features that are used here, Gmin and pH [Gmin]. The property GminN is normalised with division by the number of amino acids in a protein. In making these DH calculations of ionisable group contributions to folding energy, we modelled zero interactions in the unfolded state. This is an approximation, since average pK_a_s in the unfolded state can be perturbed from model compound values [83].

In addition to DH modelling for interactions between ionisable groups, we also used Finite Difference Poisson-Boltzmann calculations to estimate the desolvation cost or Born energy for transfer from bulk solvent to protein, of each ionised group. These calculations used protein and water relative dielectric values of 4 and 78.4, and an ionic strength of 0.15 Molar. Cumulative frequency distributions were compared for the average Born energy of each ionisable amino acid type across the range of proteins.

### Sidechain configurational entropy

The side chain entropy associated with each residue was calculated using an adaptation [97] of an earlier algorithm [98]. Then Stotal is the summed sidechain entropy for amino acids in a chain with no conformational restriction, modelling a state in which all rotamers are allowed. Sdiff is the difference between this state and the conformational restriction enforced by packing within the protein structure, i.e. a measure of the sidechain entropic penalty for protein folding. StotalN and SdiffN are the per amino acid equivalents.

The results of electrostatics and sidechain entropy calculations were collated to provide cumulative distributions of the properties of interest for each subset, giving a convenient graphical representation of their ability to separate the subsets. The significance of separation of protein sets from mesophile, moderate thermophile and hyperthermophile organisms was assessed with t-tests for the various calculated properties (Table 1). In some cases the distributions may deviate from the normal curve, so that t-test values should be used in conjunction with the plotted data to assess significance. The error bars presented in Figure 9 have been derived from a non-parametric test; bootstrap resampling.

### Surface area and patches

Accessible surface area (ASA) was calculated for all residues of all proteins in our two datasets, with total accessible area and the polar and non-polar components. This information was used to produce plots of average residue burial and to study surface patches. Non-polar patches were generated by taking each residue in turn and determining surface location (ASA > 5 Å^2^). For each surface residue a patch was defined consisting of all residues whose centre of mass lay within 2, 4, 6, 8 or 10 Å in turn of the central residue, and the non-polar ASA of that residue was added to the patch.

To study the distribution of groups bearing net charge on protein surfaces, we used a grid-based shell framework, developed previously to detect enzyme active sites [70]. On top of the surface grid we superpose spheres centred on each group likely to be ionised at neutral pH (R, K, H, D, E, N-terminus, C-terminus). All grid surface points within any sphere are assigned to 'charged', all other surface points are 'uncharged'. We then contour charged and uncharged patches. At low values of sphere radius the interstitial uncharged regions connect to form a large patch over most of the surface, and at larger values the charged regions themselves connect, isolating uncharged patches. In this latter situation we can record the sizes of protein regions that are devoid of net charge. Size is computed as the number of connected points on the grid shell. Such values can then be compared between datasets.

### The ProTherm database

Stability data (folding energy ΔG_fold _and melting temperature T_m_) for a wide range of proteins are available in the ProTherm database [71], cross-referenced to PDB structures. Only experiments with conditions near room temperature (15°C-30°C) and near neutrality (pH 5.0 to pH 8.0), for wild type monomeric proteins, were chosen. Where multiple measurements remained after this filtering, an average is taken. The PDB structures were then used to calculate properties, as for the thermophile/mesophile sets of proteins. Only 3 of the remaining proteins referenced by ProTherm had ΔG_fold _and T_m _data, whereas 100 contained ΔG_fold _data and 147 T_m _data. The calculated properties were then compared with experimental values in scatter plots.

## Abbreviations

DH, Debye-Hückel; FDPB, Finite Difference Poisson-Boltzmann; C*v*P, Charged *versus *Polar/non-charged; RCSB, Research Collaboratory for Structural Bioinformatics; PDB, Protein Data Bank; ASA, Accessible Surface Area.

## Authors' contributions

RBG and JW conceived the study, interpreted the data and wrote the final manuscript together. RBG and JW both contributed source code. RBG created the datasets and implemented most of the various computational analyses. Both authors read and approved the final manuscript.

## Supplementary Material

Additional file 1 Structure file datasets. Information as follows: For thermophile proteins in the 291 set: *PDB file and chain, Name, Organism, Organism growth temperature*, For each mesophile protein found in the 291 set: *PDB file and chain, Name, Organism, Organism growth temperature*, For each of the 102 protein pairs with E-value < 10^-2^: *E-value *For each of the 67 protein pairs with E-value < 10^-2 ^and difference in chain length ≤ 30 amino acids: *RMSD for C_α _atoms over the whole alignment (not just for a best fit subset)* Click here for file

## Figures and Tables

**Table 1:** Summary of calculated properties.

*Fig*	*Set*	*Property*	*50% cumulative value^a^*			*t-test statistic^b^*		
			
			*Meso*	*modT*	*hyperT*	*M-mT*	*mT-hT*	*M-hT*
2a	291	GminN	-0.225	-0.244	-0.300	1.04	**4.73**	**7.12**
2b	67	GminN	-0.197	-0.221	-0.305	**2.03**	**3.31**	**5.31**
3a	291	StotalN	-0.971	-0.940	-1.086	0.63	**7.83**	**9.14**
3b	67	StotalN	-0.950	-0.945	-1.168	0.98	**5.79**	**5.33**
5	291	SdiffN (0.8 VdW tol)	-0.616	-0.606	-0.613	0.31	1.03	0.94
5	291	SdiffN (1.2 VdW tol)	-0.334	-0.336	-0.326	0.97	1.95	1.34
6a	291	Contacts per atom	6.000	6.009	5.945	**2.43**	1.95	**2.06**
6b	291	Contact Order	0.301	0.342	0.322	**4.09**	**2.22**	1.92
7a	291	Born Energy (Asp)	20.19	20.27	19.72	1.27	1.09	0.03
7b	291	Born Energy (Glu)	15.47	13.57	13.87	1.51	1.53	**4.29**
7c	291	Born Energy (All)	15.83	14.65	13.97	1.40	**2.40**	**5.10**
8b	291	Polar ASA/Charged ASA	0.696	0.506	0.428	1.08	**5.77**	**12.26**
8c	291	Non-charged patch size	358.2	276.0	186.8	**2.29**	**4.90**	**8.59**
9a	291	Triple product	19.23	19.14	31.27	1.29	**7.99**	**10.89**
9b	67	Triple product	15.98	17.95	31.31	1.14	**4.90**	**6.36**

a. Values at 50% level of cumulative distributions for mesophile (Meso, M), moderate thermophile (modT, mT), and hyperthermophile (hyperT, hT)proteins.

b. Student's t-tests are employed for the 3 possible pairings, for each property/dataset. These are applied to the underlying populations of each property, and given in bold where they are different at the 95% confidence level.
